# Dr. James Dorr Clyde

**Published:** 1914-02

**Authors:** 


Dr. James Dorr Clyde, P. & S. (now Columbia) 1867, died at his home in Cherry Valley, N. Y., December 4, 1913, aged 70. He was a veteran of the Civil War.

